# Neurokinin 3 receptor antagonism as a novel treatment for menopausal hot flushes: a phase 2, randomised, double-blind, placebo-controlled trial

**DOI:** 10.1016/S0140-6736(17)30823-1

**Published:** 2017-05-06

**Authors:** Julia K Prague, Rachel E Roberts, Alexander N Comninos, Sophie Clarke, Channa N Jayasena, Zachary Nash, Chedie Doyle, Deborah A Papadopoulou, Stephen R Bloom, Pharis Mohideen, Nicholas Panay, Myra S Hunter, Johannes D Veldhuis, Lorraine C Webber, Les Huson, Waljit S Dhillo

**Affiliations:** a Department of Investigative Medicine, Imperial College London, London, UK; b Millendo Therapeutics, Inc, Ann Arbor, MI, USA; c Department of Gynaecology, Queen Charlotte's & Chelsea Hospital and Chelsea and Westminster Hospital, London, UK; d Institute of Reproductive and Developmental Biology, Imperial College London, London, UK; e Institute of Psychiatry, Psychology and Neuroscience, King's College London, London, UK; f Mayo Clinic, Rochester, MI, USA; g Scientific Partnering & Alliances, Innovative Medicines and Early Development Biotech Unit, AstraZeneca, Melbourn, UK; h Division of Experimental Medicine, Imperial College London, London, UK

## Abstract

**Background:**

Hot flushes affect 70% of menopausal women and often severely impact physical, psychosocial, sexual, and overall wellbeing. Hormone replacement therapy is effective but is not without risk. Neurokinin B signalling is increased in menopausal women, and has been implicated as an important mediator of hot flushes.

**Methods:**

This phase 2, randomised, double-blind, placebo-controlled, single-centre, crossover trial assessed the effectiveness of an oral neurokinin 3 receptor antagonist (MLE4901) on menopausal hot flushes. Eligible participants were healthy women aged 40–62 years, having seven or more hot flushes in every 24 h of which some were reported as being severe or bothersome, who had not had a menstrual period for at least 12 months, and who had not been taking any medication shown to improve menopausal flushes in the preceding 8 weeks. Participants received 4 weeks of MLE4901 (40 mg, orally, twice daily) and placebo (orally, twice daily) in random order separated by a 2 week washout period. Randomisation was completed by a central computer, and participants were allocated to treatment number in numerical order. The primary outcome was the total number of hot flushes during the final week of both treatment periods. Analyses were by intention to treat and per protocol using generalised linear mixed models and standard crossover analysis. All analyses were prespecified in the study protocol. The trial is registered at ClinicalTrials.gov, number NCT02668185.

**Findings:**

68 women were screened between Feb 3 and Oct 10, 2016, of which 37 were randomly assigned and included in an intention-to-treat analysis. 28 participants completed the trial and were included in a per-protocol analysis. MLE4901 significantly reduced the total weekly number of hot flushes by 45 percentage points (95% CI 22–67) compared with the placebo (intention-to-treat adjusted means: placebo 49·01 [95% CI 40·81–58·56] *vs* MLE4901 19·35 [15·99–23·42]; adjusted estimate of difference 29·66 [17·39–42·87], p<0·0001). Treatment was well tolerated. Three participants developed a transaminase rise (alanine aminotransferase 4·5–5·9 times the upper limit of normal) with a normal bilirubin 28 days after starting MLE4901, which normalised within 90 days.

**Interpretation:**

Treatment with a neurokinin 3 receptor antagonist (MLE4901) could be practice changing as it safely and effectively relieves hot flush symptoms without the need for oestrogen exposure. Larger scale studies of longer duration are now indicated.

**Funding:**

UK Medical Research Council and National Institute for Health Research.

## Introduction

The menopause occurs when an insufficient number of ovarian follicles remain to sustain circulating oestrogen concentrations. Consequently, menstruation ceases, fertility is lost, and in most women a cluster of symptoms become prominent, which impact physical, psychosocial, sexual, and overall wellbeing. Symptoms can be long lasting (median 7·4 years),^1^ and most women report that vasomotor symptoms (termed hot flushes or flashes or night sweats) are the most bothersome of all,^2^ with at least 10% of women reporting them as intolerable.^3^ An effective treatment is hormone replacement therapy as this artificially restores circulating oestrogen. However, hormone replacement therapy and in particular combined hormone replacement therapy is not without risk, and is contraindicated in many women due to the long-term safety concerns, including an increased risk of breast^4^ and ovarian^5^ cancers, thromboembolism,^4^ and stroke.^4^ Other treatments including selective serotonin reuptake inhibitors,^6^ gabapentin,^6^ tibolone,^6^ and cognitive behavioural therapy^7^ have been shown to have some efficacy but can cause side-effects. Herbal remedies, such as black cohosh and red clover might provide some relief but efficacy is variable between trials.^8^ It is estimated that a novel treatment for menopausal flushes could currently benefit 10 million women in the UK alone,^9^ and therefore a better understanding of the aetiology of such flushes and an associated targeted therapeutic is required.

**Research in context**

**Evidence before this study**

Hot flushes affect 70% of menopausal women and result in long-lasting symptoms, which severely impact physical, psychosocial, sexual, and overall wellbeing, in response to oestrogen withdrawal. Hormone replacement therapy is an effective treatment but is contraindicated in some women, and is not without risk in all due to long-term safety concerns, particularly an increased risk of breast cancer (though perhaps less so if it is oestrogen-only hormone replacement therapy), thromboembolism, and stroke. Alternative treatments—eg, some antidepressants and herbal remedies, have been shown to have some efficacy but not always greater than the effect of a placebo depending on the study and treatment class, and are not without side-effects too. For the past 20 years a growing body of evidence has accumulated that implicates neurokinin B (NKB) signalling (a hypothalamic neuropeptide) together with its receptor (NK3R) in the aetiology of menopausal hot flushes. We searched PubMed on Jan 6, 2017, using the keywords “neurokinin 3 receptor”, “hot flushes”, and “hot flashes” with no date or language restrictions. Of the four publications identified, one was a comprehensive review summarising all previous evidence in rodents, primates, and human post-mortem studies that shows that NKB neurons adapt in response to sex steroid deficiency, that this can be reversed by sex steroid replacement, and that NKB signalling is propagated via the hypothalamic median pre-optic nucleus (MnPO), which expresses NK3R, and receives input from, and projects to, the autonomic thermoregulatory pathway. This review included one of the other original articles identified. Of the other two, one was an original article that further supports the role of NKB signalling in the MnPO, and the other was a randomised, double-blind, placebo-controlled, crossover trial that showed that peripheral infusion of NKB intravenously induces hot flushes in healthy premenopausal women that are typical of the location, duration, and observed physiological change of those described by postmenopausal women. An original article (not identified from the search) of a genome-wide association study found that genetic variation in *TAC3R*, which is the gene that encodes NK3R, might account for the variability in experience of hot flushes reported among women.

**Added value of this study**

We provide evidence from a phase 2, randomised, placebo-controlled, crossover trial that treatment with an oral, neurokinin 3 receptor antagonist taken twice daily significantly reduces the frequency of menopausal hot flushes. Residual flushes are also significantly less severe, less bothersome, and less interfering than when taking placebo. Treatment was well tolerated and safe. The only safety caution was a mild transient rise in transaminase concentrations in a small subgroup of participants.

**Implications of all the available evidence**

The finding that pharmacological blockade of NKB signalling with an oral NK3R antagonist can significantly improve hot flush symptoms independently of any hormonal effect fits entirely with the pre-existing data, and suggests great promise for such agents as a novel therapy. Larger scale studies of longer duration will determine whether such a therapeutic approach will change future clinical practice so that the lives of those women so deeply affected by hot flushes can be transformed without the need for increased oestrogen exposure.

A hot flush is characterised by an intense feeling of heat, which often rises through the body, and intermittent activation of heat dissipation effectors, including peripheral cutaneous vasodilatation, sweating, and behavioural change, to reduce temperature.^10^ In rodents this is seen as a change in tail skin temperature secondary to vasodilatation of tail vessels, and behaviour change to increase heat loss such as grooming and hyperventilation.^10^ In human beings, a symptom diary and skin conductance monitor can reflect similar changes.^11^, ^12^

It has been known for many years that the hypothalamic-pituitary-gonadal axis regulates circulating sex steroid concentrations throughout life but it is only more recently that the role of the hypothalamic neurons containing colocalised kisspeptin, neurokinin B (NKB), and dynorphin receptors (so called KNDy neurons) has become clear.^13^ Furthermore, over the past 20 years, Rance and colleagues have contributed to a growing body of evidence in rodents,^14^, ^15^, ^16^, ^17^ primates,^18^ and post-mortem studies in human beings^19^ that the KNDy neurons, and in particular NKB and its receptor (NK3R), are implicated in the aetiology of the menopausal hot flush (summarised in a comprehensive review by Rance and colleagues^10^). Two recent publications further implicate NKB and NK3R in menopausal flushing: in a randomised, double-blind, placebo-controlled, crossover study, peripheral infusion of NKB intravenously to healthy premenopausal women induced hot flushes that were typical in location and duration to those described by postmenopausal women,^20^ and Crandall and colleagues^21^ found that genetic variation in *TACR3*, which is the gene that encodes NK3R, might account for the variability in experience of hot flushes reported among menopausal women. Such data led us to hypothesise that NKB–NK3R signalling was an important mediator of menopausal flushing, and therefore pharmacological blockade of NK3R with an oral antagonist could be a novel therapeutic target without the need for increased oestrogen exposure.

## Methods

### Study design and participants

This randomised, placebo-controlled, double-blind, two-way crossover trial was designed to assess the efficacy of an oral NK3R antagonist (MLE4901, previously termed AZD4901) in reducing the frequency of menopausal flushes in a proof-of-concept study. Eligible participants were healthy women aged 40–62 years, having seven or more hot flushes in every 24 h of which some were reported as being severe or bothersome, who had not had a menstrual period for at least 12 months, and who had not been taking any medication shown to improve menopausal flushes in the preceding 8 weeks. Full inclusion and exclusion criteria are detailed in the appendix.

Ethical approval was obtained from the West London Regional Ethics Committee (15/LO/1481), and approval was also gained from the Medicine and Healthcare products Regulatory Agency (EudraCT 2015-001553-32). All participants provided written informed consent before inclusion. The study was done in accordance with Good Clinical Practice guidelines. No changes were made to the protocol after study initiation and no interim analysis was done. An independent data monitoring committee monitored the safety of the study. Recruitment was stopped when sufficient numbers had enrolled to ensure the planned sample size was achieved, and the trial was stopped when all participants had completed the study protocol as no serious safety concerns occurred requiring early termination.

### Randomisation and masking

Randomisation was provided by Almac Clinical Services using a balanced randomisation design (1:1, MLE4901 or placebo first) made using their in-house automated Medication Number List generator to provide a Medication Number List that linked subject number to a randomised treatment group. Subject number was assigned by JKP in numerical order immediately before writing each drug prescription and taking it to the pharmacy. All authors, participants, and trial staff including the trial pharmacist and pharmacy technicians were masked until completion of the study. Unmasking only occurred once all participants had completed the study, and all their data had been entered into the electronic case record forms. To ensure safety in the case of a medical emergency, code break packs with individual scratch off panels were held by JKP, WSD, and the trial pharmacist but these were never required, and so remain unopened.

### Procedures

**Figure 1:**
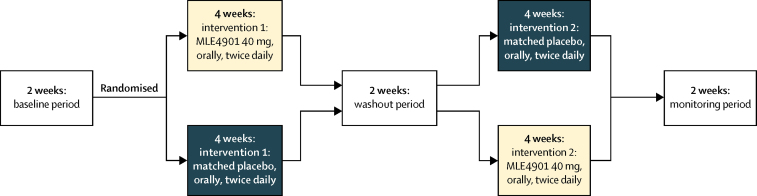
Summary of protocol Baseline period: participants underwent a 2 week period to gather baseline data on hot flush frequency, severity, bother, and interference (Hot Flash Related Daily Interference Scale). If the inclusion criteria regarding hot flush frequency and severity were met at the end of this period then they were assigned to the active phase of the study. Intervention 1 (double-blind): all participants randomly assigned to either 4 weeks of treatment with oral, twice daily 40 mg MLE4901 or exact-match placebo. Washout period: all participants underwent a 2 week washout period after intervention 1 (half-life of MLE4901 is 8·5 h). Intervention 2 (double-blind): all participants then switched to receive either 4 weeks of treatment with oral, twice daily exact-match placebo or oral, twice daily 40 mg MLE4901 depending on which intervention they received first. Monitoring period: a subsequent 2 week period to complete safety monitoring.

For the first 2 weeks of the protocol (baseline period) data were collected to establish participants' steady state, to ensure familiarity with recording symptoms and completing the questionnaires appropriately, and to enable review of the inclusion criteria regarding number and severity of flushes at the end of week 2. Participants were then randomly assigned to either 4 weeks of MLE4901 (40 mg, orally, twice daily; group 1) or exact-match placebo (group 2) followed by a 2 week washout period (half-life of MLE4901 is 8·5 h). The dose of MLE4901 was chosen following a recent study in polycystic ovarian syndrome that showed 40 mg twice daily was biologically active and safe.^22^ The participants then received 4 weeks of whichever intervention they did not receive first followed by 2 weeks of monitoring to ensure they were monitored for the same length of time after dosing irrespective of treatment assignment order (figure 1).

Participants were ambulatory, and attended one of our clinical research facilities at Imperial College NHS Trust Hospitals. They met with one of the doctors in the study team (almost always the study coordinator [JKP]) every week for 14 weeks. At each weekly visit, adverse events (according to the Common Terminology Criteria for Adverse Events version 4.0 [CTCAE]), vital signs, and liver and kidney function were recorded, and twice-daily questionnaires recording total hot flush frequency, severity, bother, interference, associated menopausal symptoms, and treatment adherence were issued and collected. Additionally, a programmed Bahr Monitor (Simplex Scientific, Middleton, WI, USA) was issued to record skin conductance during the first 48 h of each week,^12^ and serum was saved to later measure gonadotropins (see the appendix p 4 for the schedule of trial procedures).

### Outcomes

The primary outcome was total number of hot flushes during the fourth week of treatment with MLE4901 or placebo to allow adequate time for an effect to be observed.^23^ Comparison was also made between the total number of hot flushes during the fourth week of both treatment periods and the total number during the second week of the baseline period.^23^ To ensure accurate records, participants recorded their flushes in real time using either a tally chart on a piece of paper (n=34) or an application on their smartphone such as Tally Counter (Pixel Research Labs, Minneapolis-Saint Paul, MN, USA; n=3), and then collated their total number of flushes twice daily on waking to record previous overnight symptoms and before bed to record daytime symptoms.

Secondary outcomes were hot flush severity, bother, and interference, associated menopausal symptom domain scores, gonadotropin concentrations, luteinising hormone pulsatility, and number of hot flushes detected by a skin conductance monitor. Hot flush severity and bother were reported twice daily (on waking to capture previous overnight symptoms and before bed to capture daytime symptoms) using a scale that ranged from nil to severe, and from not at all to a lot, respectively. Responses were then converted into a numerical scale for data analysis as per Joffe and colleagues^23^ (severity: nil scored 1, mild scored 2, moderate scored 3, and severe scored 4; bother: not at all scored 1, a little scored 2, moderately scored 3, and a lot scored 4), and the morning and evening scores were then added to give a total daily score. Hot flush interference was recorded every evening using the Hot Flash Related Daily Interference Scale as per Carpenter,^24^ and associated menopausal symptoms were recorded every morning using the Menopause-Specific Quality of Life (MENQOL) questionnaire (which is subdivided into the four symptomatic domains: vasomotor, psychosocial, physical, and sexual) as per Hilditch and colleagues.^25^ Both of these scores were calculated as advised in the two original papers referenced but on a daily rather than a weekly or monthly basis, and then mean weekly total score was used for data analysis. To objectively measure hot flushes a skin conductance monitor (Bahr Monitor) was worn on the sternum for the first 48 h of each study week and the number of flushes was calculated by the software hot flush detection algorithm (SCM Conductance Software version 1.1.3.0, Simplex Scientific, Middleton, WI, USA).^12^ The mean weekly value for each of the secondary outcomes was compared during the fourth week of treatment with MLE4901 and placebo. Comparison was also made between the fourth week of both treatment periods and the second week of the baseline period. To assess compliance with treatment, participants also completed a modified medication adherence questionnaire twice daily, and returned their tablet bottles and any remaining tablets after both drug treatments for counting.

To measure any effect of MLE4901 on reproductive hormones, 3 mL of blood was taken from a peripheral venepuncture at each study visit to measure luteinising hormone, follicle-stimulating hormone, and oestradiol. Furthermore, to analyse luteinising hormone pulsatility a subgroup of participants based on their willingness to participate (n=13) attended our clinical research facility for three, 8 h studies. One was during the baseline period, and one during the second week of both treatment periods. During each study a 3 mL blood sample was taken every 10 min from a peripheral venous cannula that had been sited before the study start (time −30 min). All samples were left to clot for at least 30 min and then centrifuged so that the serum could be frozen for subsequent analysis. Analysis was completed in a batch when participants had finished the entire study protocol to eliminate any intra-assay variation. An automated chemiluminescent immunoassay method (Abbott Diagnostics, Maidenhead, UK) was used for analysis; for reference ranges, coefficients of variation, and analytical sensitivities see the appendix (p 5). Luteinising hormone pulsatility was determined using a blinded deconvolution method with 93% sensitivity and specificity by calculating the number and amplitude of luteinising hormone pulses, and how ordered they were.^26^

### Statistical analysis

The sample size was calculated to examine a reduction in hot flush frequency using published data from studies with similar designs.^23^, ^27^, ^28^ Allowing for a 25% reduction in hot flush frequency with placebo,^11^, ^29^ we anticipated that MLE4901 would result in a 50% reduction from a baseline of seven hot flushes per 24 h since it had been previously determined that this was the magnitude of clinical improvement needed for an effective new therapeutic for menopausal flushing (ie, at least twice the effect achieved by placebo).^23^, ^27^, ^28^ We calculated an intraparticipant hot flush frequency SD of 2·02 hot flushes per day based on raw data from a previous study by Ayers and colleagues.^7^ 27 women were required to complete the protocol (90% power, alpha 0·05, two-sided), and allowing for a 10% dropout rate^29^ we estimated that 30 women would enter the active phase of the study having met the inclusion criteria at week 2.

Data were analysed using generalised linear mixed models (as implemented in SAS PROC GLIMMIX). For the primary endpoint (weekly total hot flush count), a generalised linear mixed model with a Poisson error structure was used. For all secondary endpoints, except the total number of flushes in luteinising hormone pulsatility analysis, which used a generalised linear mixed model with a Poisson error structure, a generalised linear mixed model with gamma error structure was used since exploratory analysis indicated that the usual assumption of normality was not valid for these endpoints. For all models used, a standard crossover analysis was implemented, with period, administration sequence, and treatment as fixed effects and subject as a random effect. Tests for sequence (order) and period effects across all our models were done. All possible covariates were initially included in exploratory analyses but the only ones that were significant in each case were the baseline values of the endpoint being analysed, and so the final results reported are taken from models that included the baseline value as the only covariate. All of these models, although not assuming normally distributed errors, are entirely analogous to conventional analyses using ANCOVA and can be interpreted in the same way.

From each model, adjusted (least squares) means and differences between treatment means were estimated, together with associated 95% CIs, and a p value from a comparison of the mean values of the two treatments. For each endpoint, the percentage change from baseline was also estimated using a generalised linear mixed model (with gamma error structure) with fold-change from baseline as the analysed response, with percentage change (and 95% CI) derived by calculation from the fold-change results. This ensures that the percentage change results take into account the crossover design.

All of these analyses were prespecified as per the study protocol. The primary and all secondary endpoints were analysed using both the per-protocol and intention-to-treat analysis sets, and all p values and adjusted means for all endpoints were very similar. Accordingly, results have been presented for the intention-to-treat and per-protocol sets for the primary endpoint and for the per-protocol set only for the secondary endpoints as originally planned (though intention-to-treat analyses for the secondary outcomes as well as the per-protocol analysis for the primary outcome are reported in the appendix). To facilitate the intention-to-treat analysis of the primary endpoint, weekly total flush counts were imputed where missing by setting the count totals to be equal on both active and placebo treatment groups. However, we also did a sensitivity analysis of the primary endpoint intention-to-treat analysis using multiple imputation techniques, and this gave similar results to those reported below, with a slightly larger estimate of treatment effect. The multiple imputation method was used for all intention-to-treat analyses of secondary endpoints. All significant p values reported remained significant after adjustment for multiple testing (using the false discovery rate) procedure. The study was registered at ClinicalTrials.gov, number NCT02668185.

### Role of the funding source

This was an academic investigator initiated and led study, which was funded by the UK Medical Research Council (grant reference MR/M024954/1) and a National Institute for Health Research Professorship (to WSD, grant reference RP-2014-05-001). AstraZeneca agreed to provide their NK3R antagonist (AZD4901) and exact-matched placebo in a collaboration led by the principal investigator (WSD). The study was sponsored by the principal investigator's host institution (Imperial College London). One employee of AstraZeneca (LCW) contributed to the study design and continued to be involved in the trial management group while the study was ongoing. AstraZeneca held the background intellectual property regarding the compound. AstraZeneca subsequently exclusively licensed the compound to Millendo Therapeutics after the research protocol had been approved, and so one representative from their company (PM) also became involved in the trial management group while the study was ongoing. At this time the compound name was changed from AZD4901 to MLE4901. WSD and JKP (Imperial College London) had full access to all the data from the study and had final responsibility for the content of the report and decision to submit for publication. The data analysis was done by the trial statistician LH (Imperial College London).

## Results

**Figure 2:**
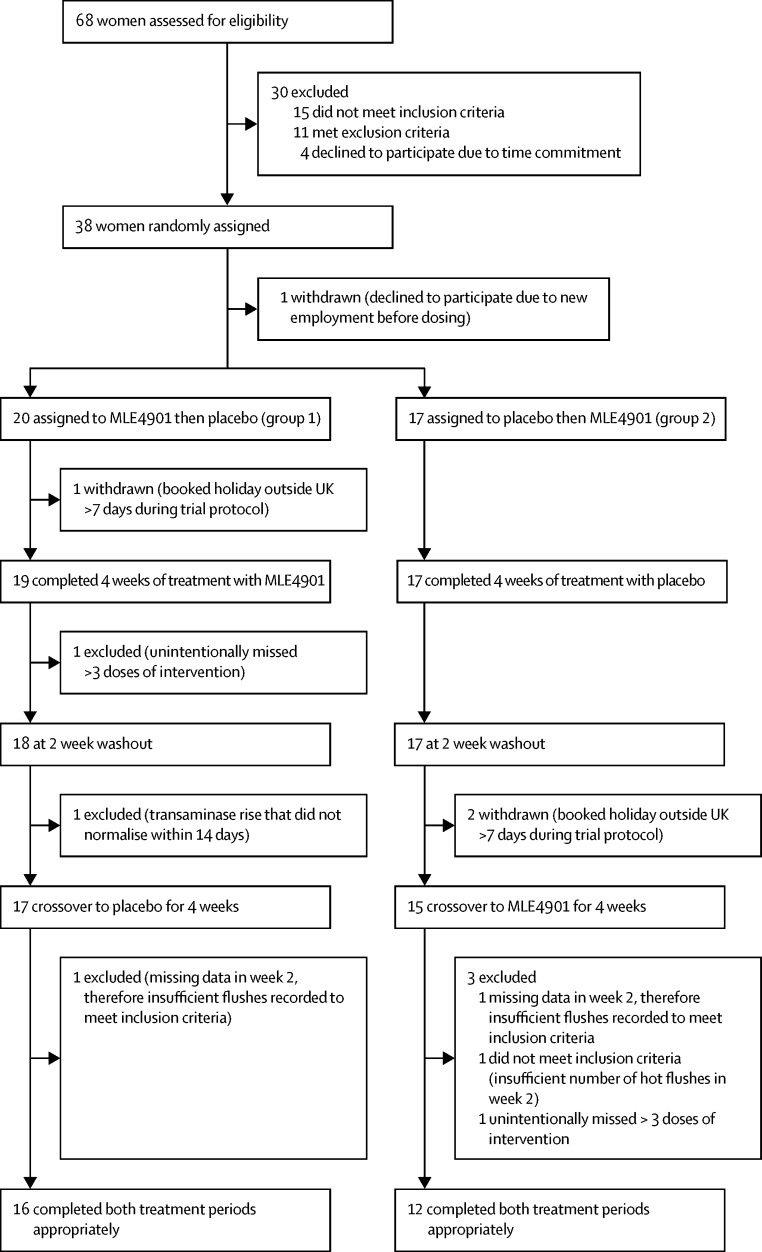
Trial profile Intention-to-treat analysis included all participants who were randomly assigned and received study medication (placebo or MLE4901; n=37). Per-protocol analysis included all participants who appropriately completed both treatment periods (n=28). See the appendix (pp 1–3) for more details about inclusion and exclusion criteria, and the number of participants affected by each.

68 women were screened by JKP between Feb 3, 2016, and Oct 10, 2016. After discussion, eight did not meet the inclusion criteria, and 11 met an exclusion criterion, and so all 19 were withdrawn (see appendix pp 1–3). Four further participants withdrew before the study began due to time commitments. Therefore, 45 participants started the trial, of which seven were identified to have not met the inclusion criteria regarding frequency or severity of flushes at the end of week 2 (as per protocol) and so were not randomly assigned and withdrawn. One participant was randomly assigned but withdrew before receiving the allocated intervention due to new employment and so was not included in any analysis. Of the remaining 37 participants who were randomly assigned and received study medication (the intention-to-treat analysis set), 28 completed the protocol (the per-protocol analysis set; age range 49–62 years and had been experiencing hot flushes for 1–16 years). Extended demographic and baseline characteristics of the 28 participants who completed the trial are summarised in table 1, and participant flow is shown in figure 2. Baseline menopausal symptom scores were similar between treatment assignment groups (table 2). Baseline mean total number of hot flushes per 24 h was 13·09 (SD 6·47) in group 1 and 12·56 (3·93) in group 2 (table 2).

**Table 1:** Demographics and baseline characteristics of the 28 women who completed the trial (per-protocol set)

		**Group 1: allocated to MLE4901 and then placebo (n=16)**	**Group 2: allocated to placebo and then MLE4901 (n=12)**
Age (years, range)		56 (50–62)	54 (49–61)
Ethnicity			
	White British	8 (50%)	9 (75%)
	White European	2 (13%)	0
	White other	1 (6%)	0
	White unspecified	0	1 (8%)
	Asian, Pakistani	1 (6%)	0
	Black Caribbean	3 (19%)	2 (17%)
	Mixed white and black Caribbean	1 (6%)	0
Body-mass index (kg/m^2^)		27·0 (4·2)	24·7 (3·7)
Blood pressure (mm Hg)			
	Normotensive	15 (94%)	10 (83%)
	Hypertensive	1 (6%)	2 (17%)
Body temperature at screening appointment (°C)		36·4 (0·4)	36·7 (0·4)
Duration of oligomenorrhoea (months)		135 (96)	96 (66)
Duration since last menstrual period (months)		119 (94)	77 (63)
Duration since hot flushes (months)		79 (47)	74 (65)
Menarche (age, years)		13 (1)	13 (2)
Regular menstrual cycles in adulthood			
	Regular	13 (81%)	10 (83%)
	Irregular	3 (19%)	2 (17%)
Gravida		2 (2)	3 (2)
Parity		1 (1)	2 (2)
Hysterectomy			
	No	11 (69%)	12 (100%)
	Hysterectomy	4 (25%)	0
	Hysterectomy and bilateral salpingo-oophorectomy	1 (6%)	0
History of hormone replacement therapy use			
	Yes	5 (31%)	1 (8%)
	No	11 (69%)	11 (92%)
History of Mirena coil use			
	Yes	2 (12%)	2 (17%)
	No	14 (88%)	10 (83%)
History of herbal remedy use of any type			
	Yes	11 (69%)	6 (50%)
	No	5 (31%)	6 (50%)
Smoking status			
	Never smoked	13 (82%)	5 (42%)
	Ex-smoker	1 (6%)	4 (33%)
	Current smoker	2 (12%)	3 (25%)
Alcohol consumption			
	≤14 units per week	16 (100%)	12 (100%)
	>14 units per week	0	0
Luteinising hormone at screening appointment (IU/L)		31·7 (11·8)	33·2 (7·3)
Follicle-stimulating hormone at screening appointment (IU/L)		70·0 (20·5)	74·9 (23·1)
Progesterone at screening appointment (nmol/L)		0·9 (0·0)	0·9 (0·0)
Oestradiol at screening appointment (pmol/L)		69 (0)	69 (0)
Prolactin at screening appointment (mU/L)		166 (47)	167 (69)
Androstenedione at screening appointment (nmol/L)		1·5 (0·8)	1·9 (1·0)
Dehydroepiandrosterone at screening appointment (μmol/L)		2·8 (2·1)	3·1 (2·0)
Sex hormone binding globulin at screening appointment (nmol/L)		61 (30)	56 (19)
Extracted testosterone at screening appointment (nmol/L)		0·7 (0·4)	0·8 (0·3)

Data are mean (SD) or n (%), unless stated otherwise.

**Table 2:** Baseline outcome data presented by treatment assignment group (per-protocol set)

		**Group 1: allocated to MLE4901 and then placebo (n=16)**	**Group 2: allocated to placebo and then MLE4901 (n=12)**
Total number of hot flushes per 24 h		13·09 (6·47)	12·56 (3·93)
Hot flush severity		5·74 (1·05)	6·36 (0·96)
Hot flush bother		5·86 (1·24)	6·14 (1·00)
Hot flush interference		48·68 (27·27)	27·38 (19·59)
MENQOL domains score			
	Vasomotor	4·25 (1·93)	4·59 (1·57)
	Psychosocial	3·50 (1·86)	2·36 (1·11)
	Physical	3·24 (1·33)	2·96 (1·32)
	Sexual	3·75 (2·54)	2·63 (1·83)
Number of flushes detected by sweat monitor per 24 h		24·86 (7·73)	27·33 (6·14)

Data are unadjusted mean (SD). MENQOL=Menopause-Specific Quality of Life questionnaire.

**Figure 3:**
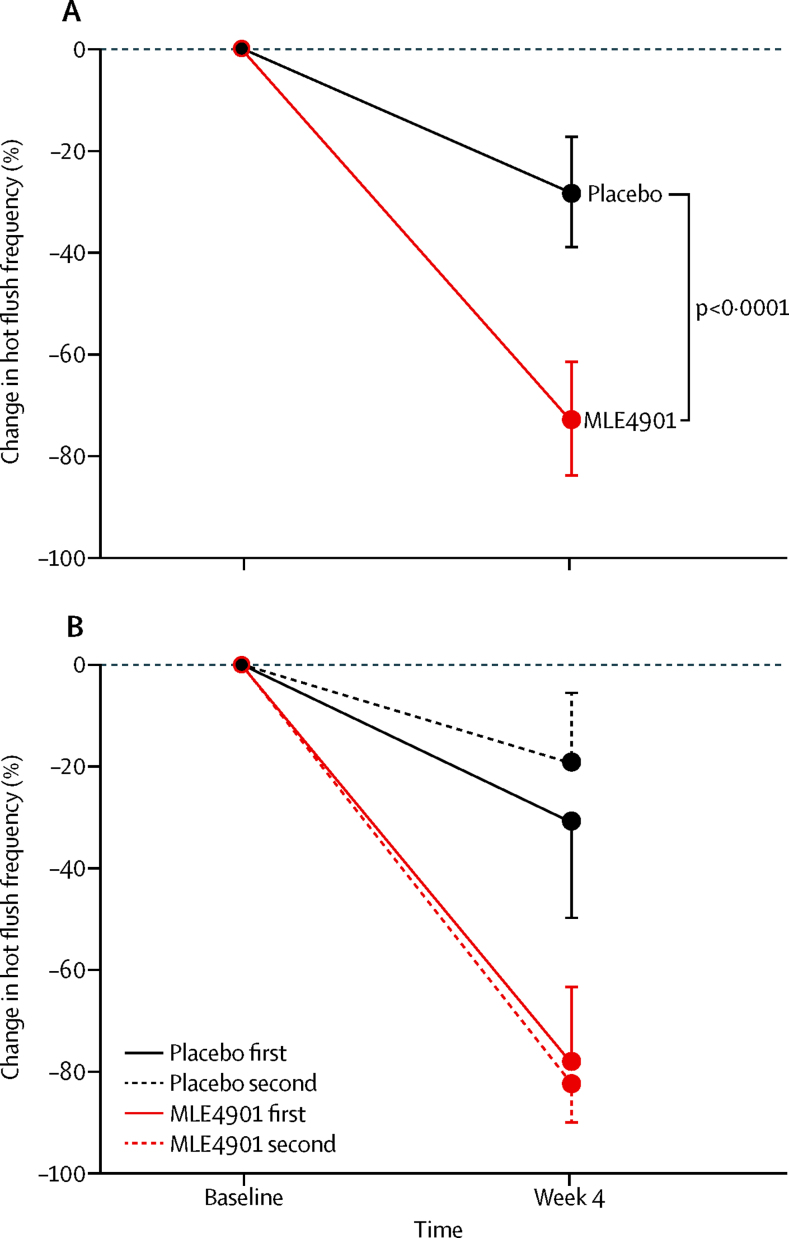
Primary endpoint ITT analysis (A) Whole group ITT analysis (n=37) irrespective of treatment assignment order using adjusted means from crossover analysis with 95% CIs: percentage change in hot flush frequency (total number of hot flushes) during the final week of the 4 week treatment period with MLE4901 and placebo compared with hot flush frequency (total number of hot flushes) during the final week of the 2 week baseline period. Statistical analysis incorporated a total of seven daily counts for each of the study weeks analysed, and is based on a crossover model including treatment and period as fixed effects, subject as a random effect (within sequence), and baseline flush count as a covariate. All other possible demographic covariates were tested in the model but none were significant and therefore all were excluded from the final model. The model used is a generalised linear model with gamma error structure. Tests for sequence (order), and period, effect across all our models confirmed neither were significant. (B) Subgroup ITT analysis (n=37) by treatment assignment group using participants' unadjusted (raw) data with 95% CIs: percentage change in hot flush frequency (total number of hot flushes) during the final week of the 4 week treatment period with MLE4901 and placebo compared with hot flush frequency (total number of hot flushes) during the final week of the 2 week baseline period depending on whether the participant received MLE4901 or placebo as the first or second intervention. ITT=intention to treat.

MLE4901 significantly reduced the total weekly number of hot flushes compared with placebo (intention-to-treat adjusted means: placebo 49·01 [95% CI 40·81–58·56] *vs* MLE4901 19·35 [15·99–23·42], p<0·0001; 45 percentage point decrease [22–67]; figure 3, table 3). The corresponding results for the per-protocol analysis were similar (placebo 59·27 [95% CI 51·52–68·17] *vs* MLE4901 16·85 [14·34–19·78], p<0·0001; 52 percentage point decrease [26–82]; appendix p 6).

**Table 3:** Primary endpoint (intention-to-treat analysis, n=37)

	**Baseline**	**On treatment**		**Difference**
		Placebo	MLE4901	
Unadjusted (raw) mean counts	84·54	62·84	25·24	..
Adjusted (least squares) means from crossover analysis (95% CI)*	..	49·01 (40·81 to 58·86)	19·35 (15·99 to 23·42)	..
Adjusted (least squares) estimate of difference (placebo – MLE4901) between treatment means (95% CI)	..	..	..	29·66 (17·39 to 42·87)
Adjusted (least squares) estimate of percentage change from baseline (95% CI)	..	−28% (−17 to −39)	−73% (−61 to −84)	..
Adjusted (least squares) estimate of percentage point difference (MLE4901 – placebo) between treatment means (95% CI)	..	..	..	−45% (−22 to −67)

Total number of hot flushes during the final week of the 4 week treatment period with MLE4901 or placebo. Comparison was also made of the total number of hot flushes during the second week of the baseline period. Statistical analysis incorporated a total of seven daily counts for each of the study weeks analysed and is based on a crossover model including treatment and period as fixed effects, subject as a random effect (within sequence), and baseline flush count as a covariate. All other possible demographic covariates were tested in the model but none were significant and therefore all were excluded from the final model. The model used is a generalised linear model with Poisson error structure for the total flush counts, and with gamma error structure to estimate percentage change from baseline for the two treatments.

* p<0·0001, comparison of treatment means.

Compared with placebo, MLE4901 also significantly reduced weekly hot flush severity (placebo 5·70 [5·09–6·38] *vs* MLE4901 3·27 [2·92–3·66], p<0·0001; 41 percentage point decrease [32–49]), bother (placebo 5·56 [4·96–6·22] *vs* MLE4901 2·92 [2·61–3·27], p<0·0001; 45 percentage point decrease [36–53]), and interference (placebo 26·48 [20·02–35·03] *vs* MLE4901 7·94 [5·76–10·95], p<0·0001; 58 percentage point decrease [40–76]; table 4). The reported subjective effect of decreased flushes by MLE4901 was confirmed by objective measurement using the skin conductance monitor and detection algorithm software (mean number of hot flushes per 24 h: placebo 26·91 [95% CI 23·16–31·27] *vs* MLE4901 16·22 [13·99–18·80], p<0·0001; 43 percentage point decrease [30–55]; table 4).

**Table 4:** Summary results of the secondary outcomes for the 28 women who completed the trial (per-protocol analysis set)

		**Placebo (adjusted mean)**	**MLE4901 (adjusted mean)**	**Adjusted estimate of percentage point difference between treatment means**	**p value comparing adjusted treatment means**
Hot flush severity		5·70 (5·09 to 6·38)	3·27 (2·92 to 3·66)	−41% (−32 to −49)	<0·0001
Hot flush bother		5·56 (4·96 to 6·22)	2·92 (2·61 to 3·27)	−45% (−36 to −53)	<0·0001
Hot flush interference		26·48 (20·02 to 35·03)	7·94 (5·76 to 10·95)	−58% (−40 to −76)	<0·0001
MENQOL domain score					
	Vasomotor	3·98 (3·38 to 4·69)	2·05 (1·74 to 2·42)	−45% (−33 to −58)	<0·0001
	Psychosocial	2·58 (2·30 to 2·90)	2·18 (1·94 to 2·45)	−15% (−5 to −25)	0·0083
	Physical	2·93 (2·63 to 3·27)	2·42 (2·17 to 2·69)	−19% (−9 to −28)	0·0002
	Sexual	2·15 (1·84 to 2·51)	1·98 (1·68 to 2·30)	−8% (5 to −23)	0·24
Number of flushes detected by sweat monitor per 24 h		26·91 (23·16 to 31·27)	16·22 (13·99 to 18·80)	−43% (−30 to −55)	<0·0001

All outcomes were compared during the final week of the 4 week treatment period with MLE4901 and exact-match placebo to allow adequate time for an effect to be observed. For all endpoints, a generalised linear model with gamma error structure was used. A standard crossover analysis was implemented, with period, administration sequence, and treatment as fixed effects and subject as a random effect. All possible baseline covariates were initially included in exploratory analyses but the only covariates that were significant, in each case, were the baseline values of the endpoint being analysed. All other demographic covariates were therefore excluded from the final model. From each model, adjusted (least squares) means and differences between means were estimated, together with associated 95% CIs. p values refer to the comparison of the mean values of the two treatments (placebo and MLE4901). MENQOL=Menopause-Specific Quality of Life questionnaire.

Mean weekly MENQOL domain scores showed that vasomotor symptoms improved when taking MLE4901 compared with placebo (placebo 3·98 [95% CI 3·38–4·69] *vs* MLE4901 2·05 [1·74–2·42], p<0·0001) as did the psychosocial domain score (placebo 2·58 [2·30–2·90] *vs* MLE4901 2·18 [1·94–2·45], p=0·0083), and physical domain score (placebo 2·93 [2·63–3·27] *vs* MLE4901 2·42 [2·17–2·69], p=0·0002; table 4). There was no significant difference between the sexual domain score when taking MLE4901 compared with when taking placebo (placebo 2·15 [95% CI 1·84–2·51) *vs* MLE4901 1·98 [1·68–2·30], p=0·24).

The magnitude of the placebo effect did vary between individuals but the variation in the treatment effect with MLE4901 was much smaller (Table 3, Table 4).

**Figure 4:**
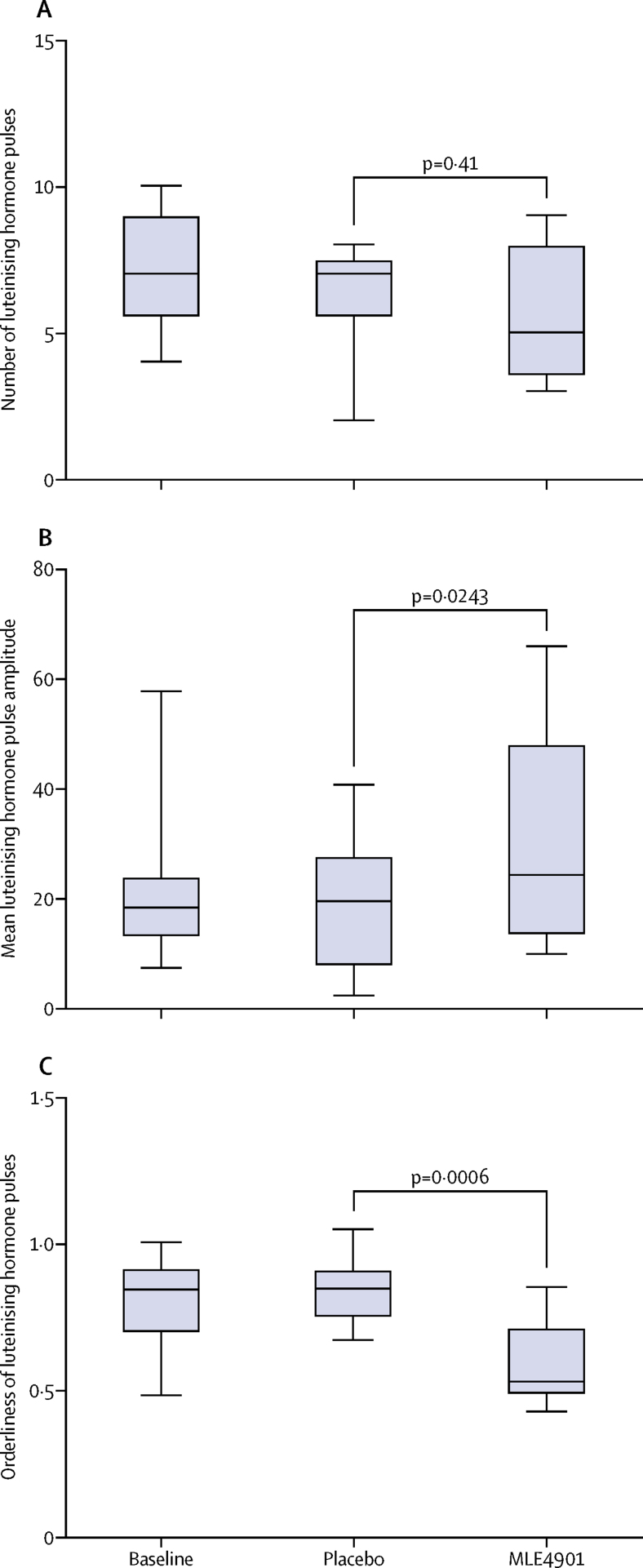
Luteinising hormone pulse analysis JDV used a blinded deconvolution method with 93% sensitivity and specificity to analyse luteinising hormone pulsatility by calculating the number of luteinising hormone pulses (A), the mean amplitude of luteinising hormone pulses (B), and the orderliness of the pulses (approximate entropy; the lower the number the more ordered the pulses are, with zero denoting perfect orderliness; C). A generalised linear model was used for analysis with a Poisson error structure for number of pulses, and with a gamma structure for mean amplitude and orderliness of luteinising hormone pulses. A standard crossover analysis was implemented, with period, administration sequence, and treatment as fixed effects and subject as a random effect. Box plots: line, median; box, IQR; whiskers extend to the extremes of the data (minimum and maximum values).

Luteinising hormone pulsatility analysis showed that treatment with MLE4901 did not change the number of luteinising hormone pulses compared with placebo (placebo 6·30 [95% CI 4·92–8·05] *vs* MLE4901 5·48 [4·21–7·13], p=0·41) but did increase the amplitude of pulses (placebo 16·16 [11·03–23·67] *vs* MLE4901 26·66 [18·20–39·05], p=0·0243) and improved their orderliness (placebo 0·84 [0·75–0·95] *vs* MLE4901 0·59 [0·53–0·66], p=0·0006; figure 4). Oestradiol remained unchanged throughout.

Data for the per-protocol analysis of the primary outcome are shown in the appendix (p 6). Data for both analysis sets of secondary outcomes are shown in the appendix (pp 7–25). Tests for sequence (order), and period, effect across all our models confirmed neither were significant (appendix p 26).

Compliance with treatment was high and both treatments were well tolerated. No serious adverse events occurred. Three participants developed a transient transaminase rise (alanine aminotransferase [ALT] greater than aspartate aminotransferase [AST]) with a normal bilirubin following treatment with MLE4901 (day 28), which remained asymptomatic, and returned to baseline in all cases. None exceeded 5·9 times the upper limit of normal (ULN) for ALT (4·5–5·9 times ULN) or 2·9 times the ULN for AST (1·7–2·9 times ULN; appendix pp 27–28). A small number of minor adverse events occurred in individual participants that were mostly unrelated to a drug effect—eg, grade 1 bruising to great toe after trauma (table 5).

**Table 5:** Adverse events

		**Baseline**	**Placebo**	**MLE4901**
**Respiratory, thoracic, and mediastinal disorders**				
Upper respiratory infection (grade 1)		0	3	6
**Nervous system disorders**				
Headache (grade 1)		0	2	3
Nervous system disorder, other, migraine (grade 2)		0	2	3
Nervous system disorder, other, S1 shingles (grade 1)		0	1	0
Sinus pain (grade 1)		0	0	1
Dysesthesia (grade 1)				
	Lip and nose	0	0	1
	Jaw and mouth	0	0	1
**Gastrointestinal disorders**				
Dry mouth (grade 1)		0	0	2
Nausea (grade 1)		0	1	2
Vomiting (grade 1)		0	0	1
Diarrhoea (grade 1)		0	0	2
Constipation (grade 1)		0	1	0
Gastro-esophageal reflux disease (grade 1)		0	0	1
**Investigations**				
ALT increased (grade 1) with normal AST		1	1	0
AST increased (grade 1) with normal ALT		1	0	1
ALT increased (grade 1) with AST increased (grade 1)		0	1	3
ALT increased (grade 2) with AST increased (grade 1)		1	0	1
ALT increased (grade 3) with AST increased (grade 1)		0	0	2
Blood bilirubin increased (grade 1)		3	0	0
Alkaline phosphatase increased (grade 1)		1	0	1
Creatinine increased (grade 1)		1	1	0
**Musculoskeletal and connective tissue disorders**				
Chest wall pain (grade 1)		0	1	0
Bruising, great toe after trauma (grade 1)		0	1	0
Neck pain, after road traffic collision (grade 2)		0	1	0
Buttock pain, traumatic (grade 1)		0	0	1
Localised oedema, fingers (grade 1)		0	0	1
Myalgia				
	Traumatic (grade 1)	0	0	1
	Atraumatic (grade 1)	0	1	1
**Skin and subcutaneous tissue disorders**				
Hypertrichosis, chin (grade 1)		0	0	1
Skin and subcutaneous tissue disorders, other, weak nails (grade 1)		0	0	1
Pruritus				
	Breast (grade 1)	0	0	1
	Hands and feet, known eczema (grade 1)	0	0	1
**Ear and labyrinth disorders**				
Ear and labyrinth disorders, other, blocked ears (grade 1)		0	0	1

Number of events recorded during the study period in all participants who received at least one dose of study medication (placebo or MLE4901; n=37). All participants were asked about adverse events at each weekly visit by the study doctor. Any reported symptom was recorded and then coded according to the Common Terminology Criteria for Adverse Events version 4.0. Counting rules were used to determine group assignment for events within the crossover trial. Group assignment: baseline, from screening through to just before the first dose of the study medication (placebo or MLE4901); placebo, from first dose of placebo to either just before the first dose of MLE4901 (if placebo received first) or through to the end of follow-up (if placebo received second); MLE4901, from first dose of MLE4901 to either just before the first dose of placebo (if MLE4901 received first) or through to the end of follow-up (if MLE4901 received second). ALT=alanine aminotransferase. AST=aspartate aminotransferase.

Compliance with the protocol was also high; only one study visit (0·83%) was missed out of 120 in total (due to suspension of the train service required for the participant to attend her weekly visit), and only three participants had suboptimal sweat monitor data (two due to a localised skin irritation caused by the topical electrode plaster and one due to losing the monitor while wearing it). Two participants unintentionally missed more than three doses of the intervention, one of which was due to bereavement, and so were both withdrawn from the study as per protocol. The dropout rate was 24% (nine of 37), which was higher than anticipated, and was mostly due to participants choosing to book a holiday outside the UK for more than 7 days during the study period.

## Discussion

In this randomised, double-blind, crossover study we have shown that an NK3R antagonist (MLE4901) significantly reduced the mean weekly number, severity, bother, and interference of hot flushes, which otherwise negatively impact a menopausal woman in her daily life. This result was achieved with a well tolerated, twice daily, oral preparation. Associated psychosocial and physical symptoms also improved with treatment (particularly those related to improved sleep such as fatigue and irritability) as otherwise disruptive night-time flushes were significantly reduced. The only safety caution was an asymptomatic rise in transaminase concentrations in a small subgroup of participants that was not associated with a rise in bilirubin. The results of ongoing and future clinical trials of MLE4901 will further explore this effect and characterise any appropriate follow-up studies required.

This study was designed as a 4 week crossover trial because it was a proof-of-concept study enabling the inclusion of fewer participants to achieve our sample calculation, a shorter trial duration,^30^ and more importantly ensured each participant acted as their own control for treatment comparisons. Baseline data generated before randomisation allowed further treatment comparisons to be made and baseline covariates to be used to reduce error variance. We allowed for a 25% reduction in hot flush frequency with placebo at 4 weeks based on previously published studies in menopausal flushing with a similar methodology to ours:^11^, ^29^ our observed rate was a 28% reduction with placebo, which therefore fits closely with the existing literature.^11^, ^29^ However, we acknowledge that there are risks of bias in crossover studies and so we avoided these by ensuring those recruited had chronic and stable flushes in the months preceding enrolment, randomisation, and blinding procedures, and the duration of the treatment and washout periods to eliminate a carryover effect. To ensure adverse events were appropriately assigned, counting rules were used to assess for treatment-emergent events. Intention-to-treat and per-protocol analyses were compared to ensure loss of participants or missing imputation methods did not distort our findings. All reported analyses conformed to the requirements of a crossover trial, and followed common practice for this study design. The objective reduction in hot flushes seen at 4 weeks using the skin conductance monitor with hot flush detection algorithm suggests that the duration of the treatment was sufficient to prove the concept. Furthermore, concordance was seen between subjective reporting and objective measurement of symptoms (45 percentage point decrease *vs* 43 percentage point decrease in hot flushes, respectively, with MLE4901 compared with placebo).

The findings from this ambulatory study should be generalisable as we placed no restriction on lifestyle, our cohort had a range of symptom duration, a quarter were not white, and some were smokers. Furthermore, no participants had received any treatment that could have improved their hot flushes for at least 8 weeks before study start, and therefore any treatment effect seen is likely to have been caused by the NK3R antagonist. Interestingly, a substantial proportion of our cohort had previously tried herbal remedies with far fewer having tried hormone replacement therapy either due to a contraindication or more commonly due to concerns regarding its use.

However, the trial is also not without limitations. The number of participants was small, the treatment duration was short, and the dropout rate was higher than anticipated. A larger and longer trial is needed to establish whether the treatment effect is long lasting in a greater number of individuals. The study was done in a single centre, and so this could be a potential source of bias. However, as a result all participants were seen and directed by the same trial doctor (JKP) during almost all of the study visits minimising variability in instructions given between participants and maximising compliance. Weekly questionnaire data regarding the primary and secondary outcomes were entered into the electronic clinical record forms by CD, RER, or ZN to reduce the likelihood of JKP observing the original data, or patterns of results that could influence opinion regarding treatment intervention when seeing the participants before the end of the study. For the same reason, data were not collated and blood samples for hormone concentrations were not analysed until participants had finished the study. Alcohol intake has previously been found to exacerbate menopausal flushes but as alcohol dependence was in our exclusion criteria all participants consumed equal to, or less than, 14 units per week. Increased body-mass index has also previously been associated with increased flushing but was not found to be an independent covariate in our analysis; this might be due to the small study size. As many of the participants were widowed, separated, or not currently sexually active any improvement in the sexual MENQOL domain by treatment with MLE4901 could have been missed as these contributory factors remained unchanged throughout the study.

Over the past 20 years a growing body of evidence has implicated NKB–NK3R signalling in the aetiology of menopausal hot flushes. By studying brain specimens at post mortem, Rance and Young^19^ initially showed that in postmenopausal women, hypothalamic neurons are hypertrophied and have increased NKB gene expression and neuronal activity compared with premenopausal women. In keeping with this, the same was found to be true in ovariectomised monkeys but, moreover, this change could be reversed by treatment with sex steroid replacement, thus suggesting this was a dynamic change in response to circulating concentrations of oestradiol as occurs in the menopause.^18^ Subsequent work in rats highlighted the importance of the hypothalamic pre-optic nucleus (MnPO) in the propagation of the NKB-mediated signal that results in hot flushes. The MnPO is a neural area that receives input from, and projects to, the autonomic thermoregulatory pathway and expresses NK3R,^14^, ^15^, ^16^, ^17^ and hence results in activation of heat dissipation effectors that characterise hot flushes. Furthermore, in a randomised, double-blind, placebo-controlled crossover study in healthy premenopausal women, peripheral infusion of NKB intravenously induced hot flushes that were typical in location, duration, and observed physiological change to those described by postmenopausal women.^20^ Additionally, Crandall and colleagues^21^ recently found that genetic variation in *TACR3*, which is the gene that encodes NK3R, might account for the variability in experience of hot flushes reported among women. Therefore, the finding that pharmacological blockade of NKB signalling with an oral NK3R antagonist can significantly improve hot flush symptoms independent of any hormonal effect fits entirely with this pre-existing data. This finding suggests great promise for such agents as a novel therapeutic target to change future clinical practice so that the lives of those women so deeply affected by hot flushes could be transformed without the need for increased oestrogen exposure. Larger scale studies of longer duration are the required next step in assessing the feasibility and likelihood of this.
