# Supplementary material for: Neurokinin 3 receptor antagonism as a novel treatment for menopausal hot flushes: a phase 2, randomised, double-blind, placebo-controlled trial
Source: Lancet. 2017 May 6;389(10081):1809–20. doi: 10.1016/S0140-6736(17)30823-1 (PMC5439024; doi:10.1016/S0140-6736(17)30823-1)
Supplement: Supplementary appendix [file mmc1.pdf]

THE LANCET

Supplementary appendix

This appendix formed part of the original submission and has been peer reviewed. We post it as supplied by the authors.

Supplement to: Prague JK, Roberts RE, Comninos AN, et al. Neurokinin 3 receptor antagonism as a novel treatment for menopausal hot flushes: a phase 2, randomised, double-blind, placebo-controlled trial. *Lancet* 2017; published online April 3.
http://dx.doi.org/10.1016/S0140-6736(17)30823-1.

SUPPLEMENTARY TABLES

Inclusion Criteria

**Inclusion criteria**	**Number of participants excluded for failing to meet this criteria**
Menopausal women (≥ 12 months since last menstrual period, or bilateral oophorectomy, or with an FSH level ≥ 20 mIU/mL and an oestradiol level <190 pmol/L in the absence of a reliable menstrual marker (hysterectomy with ovarian preservation or endometrial ablation))	2
Aged 40-62 years	0
Having seven or more flushes/24 hour period, some of which are reported as being severe or bothersome, and have been stable over the preceding months prior to enrolment	13
Not received any treatment for menopausal symptoms for the preceding eight weeks, nor receiving any regular medication for any other indication that also has evidence of improving hot flushes for the preceding eight weeks or currently – for example selective serotonin re-uptake inhibitors for depression, neuropathic pain agents for neuropathic pain	0

Exclusion criteria

**Exclusion criteria**	**Number of participants excluded for this reason**
Significant illness, as judged by the Investigator, within 2 weeks of first study visit	0
Volunteer has clinical, laboratory, or ECG evidence of uncontrolled hypertension (defined as systolic blood pressure of ≥ 160 mmHg and/or diastolic blood pressure of ≥100 mmHg); uncontrolled diabetes; or significant pulmonary, renal, hepatic, endocrine, or other systemic disease in the opinion of the Investigator	0
Participant has a history of Gilbert's syndrome, infectious hepatitis, or other significant hepatic disease (e.g. chronic hepatitis, cirrhosis, autoimmune hepatitis, primary sclerosing cholangitis, non-alcoholic steatohepatitis, or hereditary liver disease) in the opinion of the Investigator	0

Participant has a history of surgery which in the opinion of the investigator could cause malabsorption (e.g. gastric or small intestinal surgery or gastric bypass surgery or banding), or patient has a disease that causes malabsorption	0
Clinically significant abnormal ECG and/or abnormalities in ECG at screening as judged by the Investigator	1
A marked prolongation of QT/QTc interval (e.g. repeated demonstration of a QTc interval > 450 ms)	0
Confirmed history of ischaemic heart disease	0
Past (within 1 year of enrolment) or present alcohol or substance abuse	3
Has received another new chemical entity (defined as a compound which has not been approved for marketing) or has participated in any other clinical study that included drug treatment within at least 3 months of the first administration of MLE4901 in this study. The period of exclusion begins 3 months after the final dose. (Note: patients consented and screened, but not randomised in a previous study are not excluded)	1
Participant has a history of neoplastic disease within 5 years prior to signing informed consent or is currently on ongoing treatment to prevent cancer recurrence	0
Involvement in the planning and/or conduct of the study (applies to any AstraZeneca or Millendo employee and their close relatives and/or staff at the study site directly involved in the study, regardless of their role in accordance with their internal procedures)	0
Inability to understand or cooperate with the requirements of the study	0
Participant is legally or mentally incapacitated	0
Participant has significant psychiatric disease or treatment for psychiatric disease e.g. SSRI's which in the opinion of the Investigator would influence the results of the study	0
Participant has abnormal screening laboratory values as per the guidelines listed below or other clinically significant, unexplained laboratory abnormality according to the Investigator: - Aspartate aminotransferase (AST) >1·5 times upper limit normal (ULN) - Alanine aminotransferase (ALT) > 1·5 times ULN - Total bilirubin >1·5 times ULN - Serum creatinine >2·0 times ULN	0
Clinically relevant disease and abnormalities (past or present), which in the opinion of the Investigator, may either put the patient at risk to participate in this study or may influence the results of the study or the patient's ability to participate in the study	2
Participant has a history of hyperthyroidism or hypothyroidism or abnormal screening thyroid tests, as judged by the Investigator. Patients with hypothyroidism who are stable on treatment with normal thyroid function tests may be included in the study if in the opinion of the Investigator this will not influence the results of the study	2

Participant has seizures, patients with history of seizures or with conditions that increase the risk of seizures	0
Participant has a history of hypersensitivity to more than two chemical classes of drugs, including prescription and over-the-counter medications	0
Participant has taken any potent or moderate CYP3A4 or CYP2C9 inhibitors, potent or moderate CYP3A4 or CYP2C9 inducers, hormonal contraceptives, antiandrogenic drugs, or other medications for the time frame specified in the following:	
Potent and moderate CYP3A4 inhibitors, including but not limited to: cyclosporine, systemic (oral/intravenous) itraconazole, ketoconazole, fluconazole, erythromycin, clarithromycin, telithromycin, nefazodone, HIV protease inhibitors, aprepitant, verapamil, and **diltiazem** for 4 weeks prior to screening and throughout the study period	1
Potent and moderate CYP3A4 inducers, including but not limited to: rifampicin, rifabutin, carbamazepine, phenytoin, barbiturates, systemic glucocorticoids (replacements and inhaled are permitted), nevirapine, efavirenz, pioglitazone, primidone, and St. John's wort for 4 weeks prior to screening and throughout the study period	0
Potent and moderate CYP2C9 inhibitors, including but not limited to: amiodarone, fluconazole, miconazole, and oxandralone for four weeks prior to screening and throughout the study period	0
Potent and moderate CYP2C9 inducers, including but not limited to: carbamazepine and rifampicin for four weeks prior to screening and throughout the study period	0
Oral contraception, transdermal or **implantable hormonal contraception**, oestrogen, progesterone, or androgens for 8 weeks prior to screening and throughout the study period *Antiandrogenic drugs* (eg, spironolactone, any other antiandrogenic drugs), 5-α-reductase inhibitors, GnRH analogs (eg, Lupron® , any others), *ovulation induction drugs* (eg, clomiphene and any other antiestrogenic compounds, and *gonadotropins*, including all forms of FSH, LH, and human chorionic gonadotropin), and *antiprogestogens* for 12 weeks prior to screening and throughout the study period	1
Concomitant use of Statin drugs other than pitavastatin and pravastatin throughout the study	0

Supplementary Table 1 Inclusion and exclusion criteria including the number of participants who did not meet the inclusion criteria, or who met one of the exclusion criteria

	Screening		Baseline		Treatment 1				Washout		Treatment 2				Follow up	
		week	week	week	week	week	Week	week	Wee k	week	week	week	week	week	week	week
		**0**	**1**	**2**	**3**	**4**	**5**	**6**	**7**	**8**	**9**	**10**	**11**	**12**	**13**	**14**
Informed consent	x															
Review of inclusion/exclusion criteria	x															
Medical/surgical/menstrual/reproductive history	x															
Alcohol, drug abuse and smoking history	x															
Physical examination	x															
BMI	x															
Vital signs	x		x	x	x	x	x	x	x	x	x	x	x	x	x	X
ECG	x															
Record of concomitant medication	x															
Screening bloods: Haemoglobin, haematocrit, WCC, platelets, U&E's, LFT's, TFTs, prolactin, SHBG, gonadotrophins, oestradiol, androgens (androstenedione, DHEAS, extracted testosterone), progesterone, glucose, HbA1c	x															
Record AEs (CTCAE)			x	x	x	x	x	x	x	x	x	x	x	x	x	X
Reproductive hormones LH, FSH oestradiol (3ml blood) (for secondary endpoints)			x	x	x	x	x	x	x	x	x	x	x	x		
Reproductive hormones LH, FSH oestradiol measured 3 times during one week (3ml/time) (for secondary endpoints)			±		±						±					
Safety bloods: U&E's, LFT's (3ml blood)			x	x	x	x	x	x	x	x	x	x	x	x	x	X
Issue patient with questionnaires		x	x	x	x	x	x	x	x	x	x	x	x	x	x	X
Collect questionnaire completed twice daily (on waking and at bedtime) recording compliance, HF frequency, severity and bother (for secondary endpoints)			x	x	x	x	x	x	x	x	x	x	x	x		
Collect *MENQOL questionnaire completed daily on waking (for primary endpoints)			x	x	x	x	x	x	x	x	x	x	x	x		
Collect [+]HFRDIS questionnaire completed daily at bedtime (for primary endpoints)			x	x	x	x	x	x	x	x	x	x	x	x		
Download 48 hour skin conductance data (for secondary endpoints)			x	x	x	x	x	x	x	x	x	x	x	x		
8 hour sampling for LH ± FSH pulsatility[±] (max. total 144ml blood drawn) (for secondary endpoints)			±			±				±		±				±
Randomisation				x												
Issue prescription for first treatment				x												
Issue prescription for second treatment										x						

*MENQOL = Menopause Specific Quality of Life questionnaire [+]HFRDIS = Hot Flash Related Daily Interference Scale

[±]This may be performed subject to patients consent and availability (optional). NB. All treatment visits will take place at the end of the specified week

Supplementary Table 2: Schedule of trial procedures

	**Luteinising Hormone**	**Follicle Stimulating Hormone**	**Oestradiol**
Reference Range	4-14 IU/L	1·5-8 IU/L	<190 pmol/L
Intra-assay coefficient of variation	4·1%	4·1%	3·3%
Inter-assay coefficient of variation	2·7%	3·0%	3·0%
Analytical sensitivity	0·5 IU/L	0·05 IU/L	37 pmol/L

Supplementary Table 3 Laboratory analysis information for measurement of gonadotrophins and oestradiol. Samples were analysed using an automated chemiluminescent immunoassay method (Abbott Diagnostics, Maidenhead, UK).

		On treatment		
	**Baseline**	**Placebo**	**NK3R antagonist (MLE4901)**	
Unadjusted (raw) mean counts	90.14	68.32	20.32	
Adjusted (least squares) means from crossover analysis with 95% CIs		59.27 (51.52 – 68.17)	16.85 (14.34 – 19.78)	
P-value comparing treatment means				<0.0001
Adjusted (least squares) estimate of difference (placebo-MLE4901) between treatment means, with 95% CI				42.42 (31.74-53.83)
Adjusted (least squares) estimate of % change from baseline, with 95% CI		-25% (-44% - -1%)	-77% (-69% - -83%)	
Adjusted (least squares) estimate of percentage point difference (MLE4901-placebo) between treatment means, with 95% CI				-52% (-26% - -82%)

Supplementary table 4 Primary endpoint (Per protocol analysis results (n=28)): total number of hot flushes during the final week of the four week treatment period with MLE4901 or placebo. Comparison was also made to the total number of hot flushes during the second week of the baseline period. To ensure accurate records, participants recorded their flushes in real-time using either a tally chart on a piece of paper (n= 34), or an application on their smartphone such as Tally Counter (Pixel Research Labs, Inc., Minneapolis-Saint Paul, USA) (n= 3), and then collated their total number of flushes twice daily: on waking to record prior overnight symptoms and before bed to record daytime symptoms. Statistical analysis incorporated a total of seven daily counts for each of the study weeks analysed and is based on a crossover model including treatment and period as fixed effects, subject as a random effect (within sequence), and baseline flush count as a covariate. All other possible demographic covariates were tested in the model but none were statistically significant and therefore all were excluded from the final model. The model used is a generalised linear model with Poisson error structure for the total flush counts, and with gamma error structure to estimate percentage change from baseline for the two treatments.

A

		On treatment		
	**Baseline**	**Placebo**	**NK3R antagonist (MLE4901)**	
Unadjusted (raw) means	6·04	5·83	3·37	
Adjusted (least squares) means from crossover analysis with 95% CIs		5·70 (5·09 – 6·38)	3·27 (2·92 – 3·66)	
P-value comparing treatment means				<0·0001
Adjusted (least squares) estimate of difference (placebo-MLE4901) between treatment means, with 95% CI				2·43 (1·93-2·92)
Adjusted (least squares) estimate of % change from baseline, with 95% CI		-4% (-15% - +7%)	-45% (-39% - -51%)	
Adjusted (least squares) estimate of percentage point difference (MLE4901-placebo) between treatment means, with 95% CI				-41% (-32% - -49%)

B

		On treatment		
	**Baseline**	**Placebo**	**NK3R antagonist (MLE4901)**	
Unadjusted (raw) means	6.04	5.83	3.37	
Adjusted (least squares) means from crossover analysis with 95% CIs		5.78 (5.25 – 6.37)	3.38 (3.07 – 3.72)	
P-value comparing treatment means				<0.0001
Adjusted (least squares) estimate of difference (placebo-MLE4901) between treatment means, with 95% CI				2.40 (1.96-2.85)
Adjusted (least squares) estimate of % change from baseline, with 95% CI		-5% (-5% - +13%)	-44% (-39% - -50%)	
Adjusted (least squares) estimate of percentage point difference (MLE4901-placebo) between treatment means, with 95% CI				-39% (-32% - -47%)

Supplementary table 5 Hot flush severity compared during the final week of the four week treatment period with NK3R antagonist (MLE4901) and exact-match placebo. (A) Per-protocol analysis set; n=28. (B) Intention-to-

treat analysis set (multiple imputation method); n=37. Hot flush severity was recorded twice daily: on waking to capture prior overnight symptoms and before bed to capture daytime symptoms, and was graded using a scale that ranged from nil to severe. Responses were then converted in to a numerical scale for data processing and analysis whereby nil scored 1, mild scored 2, moderate scored 3 and severe scored 4. The morning and evening score was then added to give a total daily score. Analysed using a generalised linear model with gamma error structure. A standard crossover analysis was implemented, with period, administration sequence, and treatment as fixed effects, subject as a random effect, and baseline hot flush severity as a covariate.

A

		On treatment		
	**Baseline**	**Placebo**	**NK3R antagonist (MLE4901)**	
Unadjusted (raw) means	5·99	5·69	3·02	
Adjusted (least squares) means from crossover analysis with 95% CIs		5·56 (4·96 – 6·22)	2·92 (2·61 – 3·27)	
P-value comparing treatment means				<0·0001
Adjusted (least squares) estimate of difference (placebo-MLE4901) between treatment means, with 95% CI				2·64 (2·16-3·11)
Adjusted (least squares) estimate of % change from baseline, with 95% CI		-6% (-16% - +6%)	-51% (-45% - -56%)	
Adjusted (least squares) estimate of percentage point difference (MLE4901-placebo) between treatment means, with 95% CI				-45% (-36% - -53%)

B

		On treatment		
	**Baseline**	**Placebo**	**NK3R antagonist (MLE4901)**	
Unadjusted (raw) means	5.99	5.69	3.02	
Adjusted (least squares) means from crossover analysis with 95% CIs		5.66 (5.14 – 6.23)	2.96 (2.69 – 3.25)	
P-value comparing treatment means				<0.0001
Adjusted (least squares) estimate of difference (placebo-MLE4901) between treatment means, with 95% CI				2.70 (2.28-3.12)
Adjusted (least squares) estimate of % change from baseline, with 95% CI		-4% (-13% - +5%)	-50% (-45% - -55%)	
Adjusted (least squares) estimate of percentage point difference (MLE4901-placebo) between treatment means, with 95% CI				-46% (-38% - -53%)

Supplementary table 6 Hot flush bother compared during the final week of the four week treatment period with NK3R antagonist (MLE4901) and exact-match placebo. (A) Per-protocol analysis set; n=28. (B) Intention-to-

treat analysis set (multiple imputation method); n=37. Hot flush bother was recorded twice daily: on waking to capture prior overnight symptoms and before bed to capture daytime symptoms. Responses were then converted in to a numerical scale for data processing and analysis whereby not at all scored 1, a little scored 2, moderately scored 3 and a lot scored 4. The morning and evening score was then added to give a total daily score. Analysed using a generalised linear model with gamma error structure. A standard crossover analysis was implemented, with period, administration sequence, and treatment as fixed effects, subject as a random effect, and baseline hot flush bother as a covariate.

A

		On treatment		
	**Baseline**	**Placebo**	**NK3R antagonist (MLE4901)**	
Unadjusted (raw) means	37·10	31·64	12·54	
Adjusted (least squares) means from crossover analysis with 95% CIs		26·48 (20·02 – 35·03)	7·94 (5·76 – 10·95)	
P-value comparing treatment means				<0·0001
Adjusted (least squares) estimate of difference (placebo-MLE4901) between treatment means, with 95% CI				18·54 (13·35-23·72)
Adjusted (least squares) estimate of % change from baseline, with 95% CI		-14% (-36% - +15%)	-72% (-61% - -80%)	
Adjusted (least squares) estimate of percentage point difference (MLE4901-placebo) between treatment means, with 95% CI				-58% (-40% - -76%)

B

		On treatment		
	**Baseline**	**Placebo**	**NK3R antagonist (MLE4901)**	
Unadjusted (raw) means	37.10	31.64	12.54	
Adjusted (least squares) means from crossover analysis with 95% CIs		27.48 (21.58– 35.00)	8.43 (6.36 – 11.17)	
P-value comparing treatment means				<0.0001
Adjusted (least squares) estimate of difference (placebo-MLE4901) between treatment means, with 95% CI				19.05 (14.31-23.79)
Adjusted (least squares) estimate of % change from baseline, with 95% CI		-10% (-30% - +15%)	-70% (-60% - -78%)	
Adjusted (least squares) estimate of percentage point difference (MLE4901-placebo) between treatment means, with 95% CI				-60% (-44% - -76%)

Supplementary table 7 Hot flush interference compared during the final week of the four week treatment period with NK3R antagonist (MLE4901) and exact-match placebo. (A) Per-protocol analysis set; n=28. (B)

Intention-to-treat analysis set (multiple imputation method); n=37. Hot flush interference was recorded every evening using the Hot Flash Related Daily Interference Scale (HFRDIS). Analysed using a generalised linear model with gamma error structure. A standard crossover analysis was implemented, with period, administration sequence, and treatment as fixed effects, subject as a random effect, and baseline hot flush interference as a covariate.

A

		On treatment		
	**Baseline**	**Placebo**	**NK3R antagonist (MLE4901)**	
Unadjusted (raw) means	4·51	4·26	2·27	
Adjusted (least squares) means from crossover analysis with 95% CIs		3·98 (3·38 – 4·69)	2·05 (1·74 – 2·42)	
P-value comparing treatment means				<0·0001
Adjusted (least squares) estimate of difference (placebo-MLE4901) between treatment means, with 95% CI				1·93 (1·43-2·42)
Adjusted (least squares) estimate of % change from baseline, with 95% CI		-5% (-20% - +13%)	-50% (-41% - -58%)	
Adjusted (least squares) estimate of percentage point difference (MLE4901-placebo) between treatment means, with 95% CI				-45% (-33% - -58%)

B

		On treatment		
	**Baseline**	**Placebo**	**NK3R antagonist (MLE4901)**	
Unadjusted (raw) means	4.51	4.26	2.27	
Adjusted (least squares) means from crossover analysis with 95% CIs		3.85 (3.36 – 4.42)	2.10 (1.83 – 2.41)	
P-value comparing treatment means				<0.0001
Adjusted (least squares) estimate of difference (placebo-MLE4901) between treatment means, with 95% CI				1.75 (1.34-2.16)
Adjusted (least squares) estimate of % change from baseline, with 95% CI		-7% (-19% - +07%)	-48% (-41% - -55%)	
Adjusted (least squares) estimate of percentage point difference (MLE4901-placebo) between treatment means, with 95% CI				-41% (-31% - -52%)

Supplementary table 8 Vasomotor domain score of Menopause-Specific Quality of Life (MENQOL) questionnaire compared during the final week of the four week treatment period with NK3R antagonist

(MLE4901) and exact-match placebo. (A) Per-protocol analysis set; n=28. (B) Intention-to-treat analysis set (multiple imputation method); n=37. In this domain, a score was attributed to each of the following symptoms: hot flushes or flashes, night sweats, sweating. Analysed using a generalised linear model with gamma error structure. A standard crossover analysis was implemented, with period, administration sequence, and treatment as fixed effects, subject as a random effect, and baseline vasomotor domain score as a covariate.

A

		On treatment		
	**Baseline**	**Placebo**	**NK3R antagonist (MLE4901)**	
Unadjusted (raw) means	2·88	3·04	2·64	
Adjusted (least squares) means from crossover analysis with 95% CIs		2·58 (2·30 – 2·90)	2·18 (1·94 – 2·45)	
P-value comparing treatment means				0·0083
Adjusted (least squares) estimate of difference (placebo-MLE4901) between treatment means, with 95% CI				0·40 (0·13- 0·66)
Adjusted (least squares) estimate of % change from baseline, with 95% CI		4% (-7% - +15%)	-11% (-2% - -20%)	
Adjusted (least squares) estimate of percentage point difference (MLE4901-placebo) between treatment means, with 95% CI				-15% (-5% - -25%)

B

		On treatment		
	**Baseline**	**Placebo**	**NK3R antagonist (MLE4901)**	
Unadjusted (raw) means	2.88	3.04	2.64	
Adjusted (least squares) means from crossover analysis with 95% CIs		2.63 (2.38 – 2.91)	2.22 (2.01 – 2.46)	
P-value comparing treatment means				0.026
Adjusted (least squares) estimate of difference (placebo-MLE4901) between treatment means, with 95% CI				0.41 (0.18- 0.65)
Adjusted (least squares) estimate of % change from baseline, with 95% CI		2% (-6% - +11%)	-12% (-4% - -19%)	
Adjusted (least squares) estimate of percentage point difference (MLE4901-placebo) between treatment means, with 95% CI				-14% (-6% - -23%)

Supplementary table 9 Psychosocial domain score of Menopause-Specific Quality of Life (MENQOL) questionnaire compared during the final week of the four week treatment period with NK3R antagonist

(MLE4901) and exact-match placebo. (A) Per-protocol analysis set; n=28. (B) Intention-to-treat analysis set (multiple imputation method); n=37. In this domain, a score was attributed to each of the following symptoms: being dissatisfied with my personal life, feeling anxious or nervous, experiencing poor memory, accomplishing less than I used to, feeling depressed, down or blue, being impatient with other people, feelings of wanting to be alone. Analysed using a generalised linear model with gamma error structure. A standard crossover analysis was implemented, with period, administration sequence, and treatment as fixed effects, subject as a random effect, and baseline psychosocial domain score as a covariate.

A

		On treatment		
	**Baseline**	**Placebo**	**NK3R antagonist (MLE4901)**	
Unadjusted (raw) means	2·99	3·17	2·76	
Adjusted (least squares) means from crossover analysis with 95% CIs		2·93 (2·63 – 3.27)	2·42 (2·17 – 2·69)	
P-value comparing treatment means				0·0002
Adjusted (least squares) estimate of difference (placebo-MLE4901) between treatment means, with 95% CI				0·51 (0·24-0·79)
Adjusted (least squares) estimate of % change from baseline, with 95% CI		6% (-4% - +18%)	-12% (-3% - -21%)	
Adjusted (least squares) estimate of percentage point difference (MLE4901-placebo) between treatment means, with 95% CI				-19% (-9% - -28%)

B

		On treatment		
	**Baseline**	**Placebo**	**NK3R antagonist (MLE4901)**	
Unadjusted (raw) means	2.99	3.17	2.76	
Adjusted (least squares) means from crossover analysis with 95% CIs		2.97 (2.72 – 3.25)	2.45 (2.24 – 2.68)	
P-value comparing treatment means				0.0047
Adjusted (least squares) estimate of difference (placebo-MLE4901) between treatment means, with 95% CI				0.52 (0.28-0.75)
Adjusted (least squares) estimate of % change from baseline, with 95% CI		7% (-1% - +16%)	-12% (-4% - -19%)	
Adjusted (least squares) estimate of percentage point difference (MLE4901-placebo) between treatment means, with 95% CI				-19% (-10% - -26%)

Supplementary table 10 Physical domain score of Menopause-Specific Quality of Life (MENQOL) questionnaire compared during the final week of the four week treatment period with NK3R antagonist (MLE4901) and exact-

match placebo. (A) Per-protocol analysis set; n=28. (B) Intention-to-treat analysis set (multiple imputation method); n=37. In this domain, a score was attributed to each of the following symptoms: flatulence (wind) or gas pains, aching in muscles and joints, feeling tired or worn out, difficulty sleeping, aches in back of head or neck, decrease in physical strength, decrease in stamina, feeling a lack of energy, drying skin, weight gain, increased facial hair, changes in appearance, texture, or tone of your skin, feeling bloated, low backache, frequent urination, involuntary urination when laughing or coughing. Analysed using a generalised linear model with gamma error structure. A standard crossover analysis was implemented, with period, administration sequence, and treatment as fixed effects, subject as a random effect and baseline physical domain score as a covariate.

A

		On treatment		
	**Baseline**	**Placebo**	**NK3R antagonist (MLE4901)**	
Unadjusted (raw) means	2·80	2·83	2·58	
Adjusted (least squares) means from crossover analysis with 95% CIs		2·15 (1·84 – 2·51)	1·98 (1·68 – 2·30)	
P-value comparing treatment means				0·24
Adjusted (least squares) estimate of difference (placebo-MLE4901) between treatment means, with 95% CI				0·17 (-0.12 - +0.48)
Adjusted (least squares) estimate of % change from baseline, with 95% CI		1% (-13% - +17%)	-7% (-20% - +8%)	
Adjusted (least squares) estimate of percentage point difference (MLE4901-placebo) between treatment means, with 95% CI				-8% (+5% - -23%)

B

		On treatment		
	**Baseline**	**Placebo**	**NK3R antagonist (MLE4901)**	
Unadjusted (raw) means	2.80	2.83	2.58	
Adjusted (least squares) means from crossover analysis with 95% CIs		2.07 (1.79 – 2.39)	1.90 (1.64 – 2.19)	
P-value comparing treatment means				0.3552
Adjusted (least squares) estimate of difference (placebo-MLE4901) between treatment means, with 95% CI				0.17 (-0.11 - +0.45)
Adjusted (least squares) estimate of % change from baseline, with 95% CI		6% (-8% - +23%)	-1% (-14% - +14%)	
Adjusted (least squares) estimate of percentage point difference (MLE4901-placebo) between treatment means, with 95% CI				-7% (-5% - 22%)

Supplementary table 11 Sexual domain score of Menopause-Specific Quality of Life (MENQOL) questionnaire compared during the final week of the four week treatment period with NK3R antagonist (MLE4901) and exact-

match placebo. (A) Per-protocol analysis set; n=28. (B) Intention-to-treat analysis set (multiple imputation method); n=37. In this domain, a score was attributed to each of the following symptoms: change in sexual desire, vaginal dryness during sexual intercourse, avoiding intimacy. Analysed using a generalised linear model with gamma error structure. A standard crossover analysis was implemented, with period, administration sequence, and treatment as fixed effects, subject as a random effect and baseline sexual domain score as a covariate.

		On treatment		
	**Baseline**	**Placebo**	**NK3R antagonist (MLE4901)**	
Unadjusted (raw) means	25·20	26·11	17·07	
Adjusted (least squares) means from crossover analysis with 95% CIs		26·91 (23·16 – 31·27)	16·22 (13·99 – 18·80	
P-value comparing treatment means				< 0·0001
Adjusted (least squares) estimate of difference (placebo-MLE4901) between treatment means, with 95% CI				10·69 (7·56 – 13·82)
Adjusted (least squares) estimate of % change from baseline, with 95% CI		+8% (-7% - +26%)	-35% (-24% - -44%)	
Adjusted (least squares) estimate of percentage point difference (MLE4901-placebo) between treatment means, with 95% CI				-43% (-30% - -55%)

B

		On treatment		
	**Baseline**	**Placebo**	**NK3R antagonist (MLE4901)**	
Unadjusted (raw) means	25.20	26.11	17.07	
Adjusted (least squares) means from crossover analysis with 95% CIs		24.94 (22.02 – 28.26)	16.53 (14.59 – 18.72	
P-value comparing treatment means				0.0002
Adjusted (least squares) estimate of difference (placebo-MLE4901) between treatment means, with 95% CI				10.69 (7.56 – 13.82)
Adjusted (least squares) estimate of % change from baseline, with 95% CI		+4% (-8% - +22%)	-31% (-22% - -39%)	
Adjusted (least squares) estimate of percentage point difference (MLE4901-placebo) between treatment means, with 95% CI				-35% (-14% - -57%)

Supplementary table 12 Mean number of hot flushes/24 hours detected by skin conductance monitor and hot flush algorithm software compared during the final week of the four week treatment period with NK3R

antagonist (MLE4901) and exact-match placebo. (A) Per-protocol analysis set; n=28. (B) Intention-to-treat analysis set (multiple imputation method); n=37. To objectively measure hot flushes a skin conductance monitor (Bahr monitor™, Simplex Scientific LLC, Wisconsin, USA) was worn on the sternum for the first 48 hours of each week and the number of flushes was calculated by the software hot flush detection algorithm (SCM Conductance Software v1.1.3.0, Simplex Scientific LLC, Wisconsin, USA). Analysed using a generalised linear model with gamma error structure. A standard crossover analysis was implemented, with period, administration sequence, and treatment as fixed effects, subject as a random effect, and baseline average number of hot flushes/24 hours detected by the monitor as a covariate.

		Treatment		
	**Baseline**	**Placebo**	**NK3R antagonist (MLE4901)**	
Unadjusted (raw) mean counts	7·15	6·38	5·54	
Adjusted (least squares) means from crossover analysis with 95% CIs		6.30 (4·92 – 8·05)	5·48 (4·21 – 7·13)	
P-value comparing treatment means				0·4113
Adjusted (least squares) estimate of difference (placebo-MLE4901) between treatment means, with 95% CI				0·82 (-0·51 – 2·15)
Adjusted (least squares) estimate of % change from baseline, with 95% CI		-9% (-9% - +14%)	-20% (-36% - 0%)	
Adjusted (least squares) estimate of percentage point difference (MLE4901-placebo) between treatment means, with 95% CI				-11% (+6- -29%)

Supplementary table 13 LH pulse analysis: number of pulses LH pulsatility was analysed in a subgroup of participants based on their willingness to participate (n=13). All thirteen participants attended our clinical research facility for three, eight-hour studies (one during the baseline period, and one during each of the second weeks of each drug period) during which a 3 millilitre blood sample was taken every ten minutes (from time 0 to 480 minutes) from a peripheral venous cannula that had been sited prior to the study start (time -30 minutes) for gonadotrophins and oestradiol. An automated chemiluminescent immunoassay method (Abbott Diagnostics) was used for analysis. JDV used a blinded deconvolution method with 93% sensitivity and specificity to analyse LH pulsatility by calculating the number of LH pulses. A generalised linear model was used for analysis with a Poisson error structure. A standard crossover analysis was implemented, with period, administration sequence, and treatment as fixed effects, subject as a random effect, and baseline number of LH pulses as a covariate.

		On treatment		
	**Baseline**	**Placebo**	**NK3R antagonist (MLE4901)**	
Unadjusted (raw) means	21·06	19·10	30·71	
Adjusted (least squares) means from crossover analysis with 95% CIs		16.16 (11·03 – 23·67)	26·66 (18·20 – 39·05)	
P-value comparing treatment means				0·0243
Adjusted (least squares) estimate of difference (placebo-MLE4901) between treatment means, with 95% CI				-10·50 (-17·99 - -3·00)
Adjusted (least squares) estimate of % change from baseline, with 95% CI		-14% (-41% - +26%)	46% (0% - 113%)	
Adjusted (least squares) estimate of percentage point difference (MLE4901-placebo) between treatment means, with 95% CI				60% (19% - 101%)

Supplementary table 14 LH pulse analysis: mean amplitude of pulses LH pulsatility was analysed in a subgroup of participants based on their willingness to participate (n=13). All thirteen participants attended our clinical research facility for three, eight-hour studies (one during the baseline period, and one during each of the second weeks of each drug period) during which a 3 millilitre blood sample was taken every ten minutes (from time 0 to 480 minutes) from a peripheral venous cannula that had been sited prior to the study start (time -30 minutes) for gonadotrophins and oestradiol. An automated chemiluminescent immunoassay method (Abbott Diagnostics) was used for analysis. JDV used a blinded deconvolution method with 93% sensitivity and specificity to analyse LH pulsatility by calculating the mean amplitude of LH pulses. A generalised linear model was used for analysis with a gamma error structure. A standard crossover analysis was implemented, with period, administration sequence, and treatment as fixed effects, subject as a random effect, and baseline amplitude of LH pulses as a covariate.

		On treatment		
	**Baseline**	**Placebo**	**NK3R antagonist (MLE4901)**	
Unadjusted (raw) means	0·81	0·84	0·59	
Adjusted (least squares) means from crossover analysis with 95% CIs		0·84 (0·75 – 0·95)	0·59 (0·53 – 0·66)	
P-value comparing treatment means				0·0006
Adjusted (least squares) estimate of difference (placebo-MLE4901) between treatment means, with 95% CI				0·25 (0·18 - 0·33)
Adjusted (least squares) estimate of % change from baseline, with 95% CI		7% (-6% - 20%)	-25% (-34% - -15%)	
Adjusted (least squares) estimate of percentage point difference (MLE4901-placebo) between treatment means, with 95% CI				-32% (-22% - -42%)

Supplementary table 15 LH pulse analysis: orderliness of pulses LH pulsatility was analysed in a subgroup of participants based on their willingness to participate (n=13). All thirteen participants attended our clinical research facility for three, eight-hour studies (one during the baseline period, and one during each of the second weeks of each drug period) during which a 3 millilitre blood sample was taken every ten minutes (from time 0 to 480 minutes) from a peripheral venous cannula that had been sited prior to the study start (time -30 minutes) for gonadotrophins and oestradiol. An automated chemiluminescent immunoassay method (Abbott Diagnostics) was used for analysis. JDV used a blinded deconvolution method with 93% sensitivity and specificity to analyse LH pulsatility by calculating the orderliness of pulses (approximate entropy). The lower the number the more ordered the pulses are, with zero denoting perfect orderliness. A generalised linear model was used for analysis with a gamma error structure. A standard crossover analysis was implemented, with period, administration sequence, and treatment as fixed effects, subject as a random effect, and baseline orderliness of LH pulses as a covariate.

Outcome	p-value
Total number of hot flushes/24 hour period	
PP analysis	0.41
ITT analysis	0.65
Hot flush severity	
PP analysis	0.41
ITT analysis	0.41
Hot flush bother PP analysis	
PP analysis	0.41
ITT analysis	0.24
Hot flush interference PP analysis	
PP analysis	0.24
ITT analysis	0.41
MENQOL domains score	
Vasomotor	
PP analysis	0.74
ITT analysis	0.59
Psychosocial	
PP analysis	0.77
ITT analysis	0.77
Physical	
PP analysis	0.84
ITT analysis	0.77
Sexual	
PP analysis	0.89
ITT analysis	0.91
Number of flushes recorded by sweat monitor/24 hour period	
PP analysis	0.77
ITT analysis	0.59
LH pulse analysis (subset of participants)	
Number of pulses	0.59
Amplitude of pulses	0.71
Orderliness of pulses	0.82

Supplementary table 16 P-values derived from the sequence effect tests across all per-protocol (PP) and ITT (intention-to-treat) analyses.

Participant number	Group 1: Allocated to MLE4901 then placebo (n= 20)			Group 2: Allocated to placebo then MLE4901 (n= 17)		
	Baseline (n= 20)	Placebo (n= 18)	MLE4901 (n= 20)	Baseline (n= 17)	Placebo (n= 17)	MLE4901 (n= 15)
1	0	0	0	0	0	0
2	0	N/A	3/1[a]	0	1/1[g]	0
3	0	0	1/1[b]	0	0	0
4	0	0	0	0	0	0
5	0	0	0/1[c]	0	0	N/A
6	0	0	0	0	0	N/A
7	0	N/A	0	0	0	0
8	0	0	0	0	0	0
9	0	0	0	2/1[h]	(1*/0)[h]	3/1*[h]
10	0	0	0	0	0	0
11	0	0	0	0	0	0
12	1/0[d]	0	0	0	0	2/1[i]
13	0	0	0	0	0	0
14	0	0	0	0/1[j]	0	0
15	0	0	0	0	0	0
16	0	0	1/1[e]	0	0	0
17	0	0	1/1[f]	0	0	0
18	0	0	0	-	-	-
19	0	0	0	-	-	-
20	0	0	0	-	-	-

Supplementary Table 17 Adverse events: transaminase elevations by treatment and CTCAE grade in all participants who received at least a single dose of study medication (placebo or MLE4901) (n= 37). Transaminase levels were checked each week. Any abnormal result was coded according to the Common Terminology Criteria for Adverse Events v4·0 (CTCAE). Counting rules were used to determine group assignment for events within the crossover trial. Group assignment: Baseline: from screening through to just before the first dose of the study medication (placebo or MLE4901); Placebo: from first dose of placebo to either just before the first dose of MLE4901 (if placebo received first) or through to the end of follow-up (if placebo received second); MLE4901: from first dose of MLE4901 to either just before the first dose of placebo (if MLE4901 received first) or through to the end of follow-up (if MLE4901 received second). Number in the table represents CTCAE Grade (alanine aminotransferase (ALT)/aspartate aminotransferase (AST)). Normal ranges were as follows: ALT: 0-40 (iµ/L); AST 0-40 (iµ/L); Bilirubin (0-21 µmol/L). Intra-assay coefficient of variation: ALT: at mean concentration of 27, 4.19%; AST at mean concentration of 37, 2.1%; bilirubin at mean concentration of 12, 2.3%. Individual abnormal results were as follows (upper limit of normal (ULN)):

[a] week 1: ALT 23, AST 19, bilirubin 7; 28 days after starting MLE4901 ALT 121, AST 88, bilirubin 10; increased further seven days later ALT 235 (5.9x ULN), AST 108 (2.7x ULN), bilirubin 12; normalised 42 days after stopping MLE4901 ALT 32, AST 24, bilirubin 9

[b] week 1: ALT 22, AST 31, bilirubin 26; week 2 ALT 44 (1.1x ULN), AST 29, 13; normalised 7 days later before starting MLE4901 ALT 24, AST 26, bilirubin 17

[c] week 1: ALT 21, AST 25, bilirubin 8; seven days after finishing MLE4901 ALT 26, AST 42 (1.05x ULN), bilirubin 7, normalised seven days later ALT 24, AST 27, bilirubin 6

[d] week 1: ALT 22, AST 31, bilirubin 26; week 2 ALT 44, (1.1x ULN) AST 29, bilirubin 13; normalised seven days later before starting MLE4901 ALT 24, AST 26, bilirubin 17

[e] week 1: ALT 12, AST 20, bilirubin 15; seven days after finishing the 28 day treatment period with MLE4901 ALT 93 (2.3x ULN), AST 48 (1.2x ULN), bilirubin 11; normalised 14 days later ALT 17, AST 19, bilirubin 9

[f] week 1: ALT 21, AST 30, bilirubin 5; seven days after finishing the 28 day treatment period with MLE4901 ALT 59 (1.5x ULN), AST 49 (1.2x ULN), bilirubin 6; normalised 14 days later ALT 29, AST 33, bilirubin 5
[g] week 1: ALT 35, AST 33, bilirubin 6; 21 days after starting placebo ALT 47, AST 43, bilirubin 10; increased further seven days later to ALT 52 (1.3x ULN), AST 41 (1.03x ULN), bilirubin 10; normalised 14 days later ALT 40, AST 40, bilirubin 11 before starting MLE4901
[h] week 1: ALT 19, AST 23, bilirubin 5, week 2; ALT 129 (3.2x ULN), AST 51 (1.3x ULN), bilirubin 7; normalised seven days later; then seven days after stopping placebo ALT 53, AST 33, bilirubin 7; normalised seven days later; 28 days after starting MLE4901 ALT 232 (5.8x ULN), AST 118 (2.9x ULN), bilirubin 6; normalised 56 days after stopping MLE4901 ALT 47 (different assay as done locally – normal range 0-50 iμ/L))
[*] not counted as treatment emergent since already present at baseline
[i] week 1: ALT 24, AST 26, bilirubin 24; 28 days after starting MLE4901 ALT 100, AST 62, bilirubin 14; increased further seven days later ALT 179 (4.5x ULN), AST 67 (1.7x ULN), bilirubin 13; 28 days after stopping MLE4901 ALT 43, AST 28, bilirubin 16; fourteen days later rechecked after an episode of viral gastroenteritis ALT 60, AST 36, bilirubin 14; normalised 90 days after stopping MLE4901 ALT 30, AST 25, bilirubin 16
[j] week 1: ALT 10, AST 34, bilirubin 5; week 2 ALT 12, AST 44, bilirubin 6; normalised seven days later prior to starting placebo ALT 12, AST 34, bilirubin 4
